# Supplementary material for: KAT: a K-mer analysis toolkit to quality control NGS datasets and genome assemblies
Source: Bioinformatics. 2016 Nov 28;33(4):574–6. doi: 10.1093/bioinformatics/btw663 (PMC5408915; doi:10.1093/bioinformatics/btw663)
Supplement: Supplementary Data [file btw663_supp.pdf]

The Kmer Analysis Toolkit - Supplementary Information

Daniel Mapleson, Gonzalo Garcia Accinelli, George Kettleborough,
Jonathan Wright, and Bernardo J. Clavijo.

October 6, 2016

1 Tools

Table 1 lists the tools currently contained in KAT as of V2.1. We go on to describe those tools later in this section, however for a more up-to-date and detailed description of the tools please refer to the online manual: `http://kat.readthedocs.io/en/latest/`

hist	Creates a k-mer spectrum from a sequence set
gcp	Compares a k-mer coverage spectrum with k-mer GC spectrum
comp	Compares two k-mer spectra
sect	Marks k-mer frequencies from a separate dataset along a list of sequences
filter	Isolation of k-mers and sequences based on k-mer coverage and GC content

Table 1: A list of tools within KAT v2.1.0

1.1 K-mer spectrum histogram

The KAT *hist* tool creates a k-mer spectrum from a sequence set represented as a histogram showing the number of k-mers observed at each frequency. The tool outputs both a flat text file containing the k-mer spectrum, as well as a plot. This tool is similar to the jellyfish *histo* tool, and can be used to estimate of k-mers deriving from errors, assess sequencing bias, gauge completeness of genome sequencing, identify genomic properties, such as levels of heterozygosity, homozygosity, karyotype and repeat content. The tool can also help to identify contamination detection through highlighting deviation from expected frequencies.

1.2 GC vs Coverage

The KAT *gcp* tool also produces a k-mer spectrum and a GC count per distinct k-mer for a single input set. Therefore instead of a histogram, a heatmap is generated, with k-mer GC count on one dimension and k-mer frequency in the other, and the number of distinct k-mers are represented by intensity of colour. By incorporating GC information users are better able to identify unexpected content, such as sequencing biases or contamination, than through the standard k-mer spectrum alone. See the KAT online documentation for a more complete discussion of the *gcp* tool: `http://kat.readthedocs.io/en/latest/using.html#gcp`. Also see how the *gcp* tool can be used to help identify contaminants here: `http://kat.readthedocs.io/en/latest/walkthrough.html#contamination-detection-and-extraction`.

1.3 K-mer spectra comparison

The KAT *comp* tool creates and compares spectra from two datasets, recording the intersection of distinct k-mer frequencies between each set, producing a 2D matrix, with frequencies along each dimension and distinct k-mer counts in the cells. The matrix can be used in several ways. The first way is to visualise the matrix as a heatmap, showing the intersection of spectra highlighting similarities and differences between the datasets, or as a set of histograms, highlighting exclusive and shared content. Also, if the first input is a WGS dataset and the second a genome assembly of that data, then a stacked histogram maybe produced, which has proven useful for assembly validation (see main paper Section 2.1 and SI Section 5 for more details).

1.4 Sequence coverage profiler

The KAT *sect* tool, marks the k-mer frequency, and optionally GC counts, against a list of sequences, producing summary statistics and FastA style output files detailing k-mer frequencies and GC content across each target sequence. This tool is useful for contamination detection at a whole file and per sequence level, it can identify and separate distinct and repetitive regions within a genome assembly. See the KAT online documentation for a more complete section of the *sect* tool: `http://kat.readthedocs.io/en/latest/using.html#sect`.

1.5 Filtering tools

KAT contains two tools for filtering content based on k-mers. The first allows a user to produce a subset of a k-mer hash by limiting GC and frequency values, the second performs sequence filtering based on the presence or absence of a k-mer in the sequence. These tools are particularly useful for isolating contaminants or organelles from larger NGS datasets. See the KAT online documentation for a more complete section of the *filter* tools: `http://kat.readthedocs.io/en/latest/using.html#filtering-tools`. Also see how the *filter* tools can be used to extract contaminants here: `http://kat.readthedocs.io/en/latest/walkthrough.html#contamination-detection-and-extraction`.

2 Choosing a *k* value for analysis

The choice of k can affect KAT analyses. Larger values of k allow of a larger set of different k-mers. In the context of Whole Genome Sequencing (WGS), this translates to each k-mer representing a progressively more distinct region of the genome as k increases, which increases the number of elements in the first and more informative components of the spectrum. However, analyses with large k are more sensitive to sequencing errors (there is an increased likelihood that each k-mer will contain an error), generate fewer k-mers overall (each read generates $length + 1 - k$ kmers) and will require additional system memory (see Supplementary Figure 4).

The trade off in the choice of k is illustrated in Supplementary Figure 1 within the context of assembly analysis and validation (see SI Section 5 for an explanation of how to interpret the stacked histogram plots). The plot shows results for varying k in the assemblies from the Main Text's Figure 1a and an *A. thaliana* assembly. As k grows, more content is contained in the homozygous and heterozygous distributions but their centres get closer, eventually making it difficult to distinguish between them. Also, as k grows, more k-mers are affected by small differences in between haplotypes (arguably mostly SNPs) and that alters the relative abundance of elements between the heterozygous and homozygous distributions.

In general, we find smaller and less repetitive genomes can be analysed using lower k values, but larger and more repetitive genomes benefit from larger k values, given sufficient sequencing coverage and system memory. In practice, we find a k value between 17 and 63 provides enough definition to be informative. By default, KAT uses a k value of 27, which is a reasonable starting point for most cases, including typical mammalian genomes and does not require excessive memory.

3 Comparison with other reference-free k-mer analysis software

SGA PreQC(Simpson, 2014) calculates metrics about the quality of the reads such as per-base error rates, paired-end fragment-size distributions and coverage in the absence of a reference genome. Additionally, the software estimates genome characteristics, such as repeat content and heterozygosity that are key determinants of assembly difficulty. However, the tool is not designed for pairwise comparison of k-mer spectra, which is one of the strengths of KAT.

kPAL (Anvar *et al.*, 2014) also facilitates alignment-free quality assessment of sequence data. In addition, it supports pairwise comparisons of k-mer spectra enabling detection of high-duplication rates, library chimeras, contamination and differences between sequencing protocols. kPAL is however limited in that it does not scale well to large datasets. Memory usage and runtimes are high compared to KAT. In addition it is limited to exploring relatively small k values, as shown in section 4.2.

FastQC (`http://www.bioinformatics.babraham.ac.uk/projects/fastqc/`) is a popular tool for QC of NGS datasets that also has a k-mer analysis component embedded within it. However, FastQC uses small k-mers to detect overrepresented sequences within datasets, which is primarily used for detection of adaptors or other sequences which are not biological in nature and likely to stem from sequencing problems. FastQC also does not create k-mer spectra and has no functionality for comparing datasets.

4 Runtime performance

All tests in this section were performed using KAT V2.1.0. Unless otherwise noted the Arabidopsis thaliana WGS dataset ERR409722 was used as input. This dataset contains 29,888,822 100 bp paired end reads, making up a total of approximately 6 Gbp (giga base pairs) and 15GB (gigabytes) of data. Unless otherwise specified, all tests were performed on a machine with 128GB and 4 AMD Opteron(tm) 6134 Processors, connected to an Isilon scale-out NAS.

4.1 K-mer counting compared to Jellyfish2

KAT leverages a modified version of the Jellyfish2 library to perform k-mer counting. The changes implemented means KAT avoids filtering and dumping k-mer hashes to storage (unless requested to by the user). To show the effects the modifications have on wall-clock runtime, two plots are produced showing effect of thread count on two different types of storage system. Using Isilon scale-out NAS (Supplementary Figure 2) little improvement is gained due to the capacity of the storage system to handle multiple parallel IO operations, however on more conventional SSD storage (Supplementary Figure 3) runtimes are approximately 20% faster on average for KAT for this environment. The Isilon system is able to hide the time required to write the 1.9GB of ERR409722 k-27 hash, by performing the operations in parallel with reading and counting. Because the data structures used

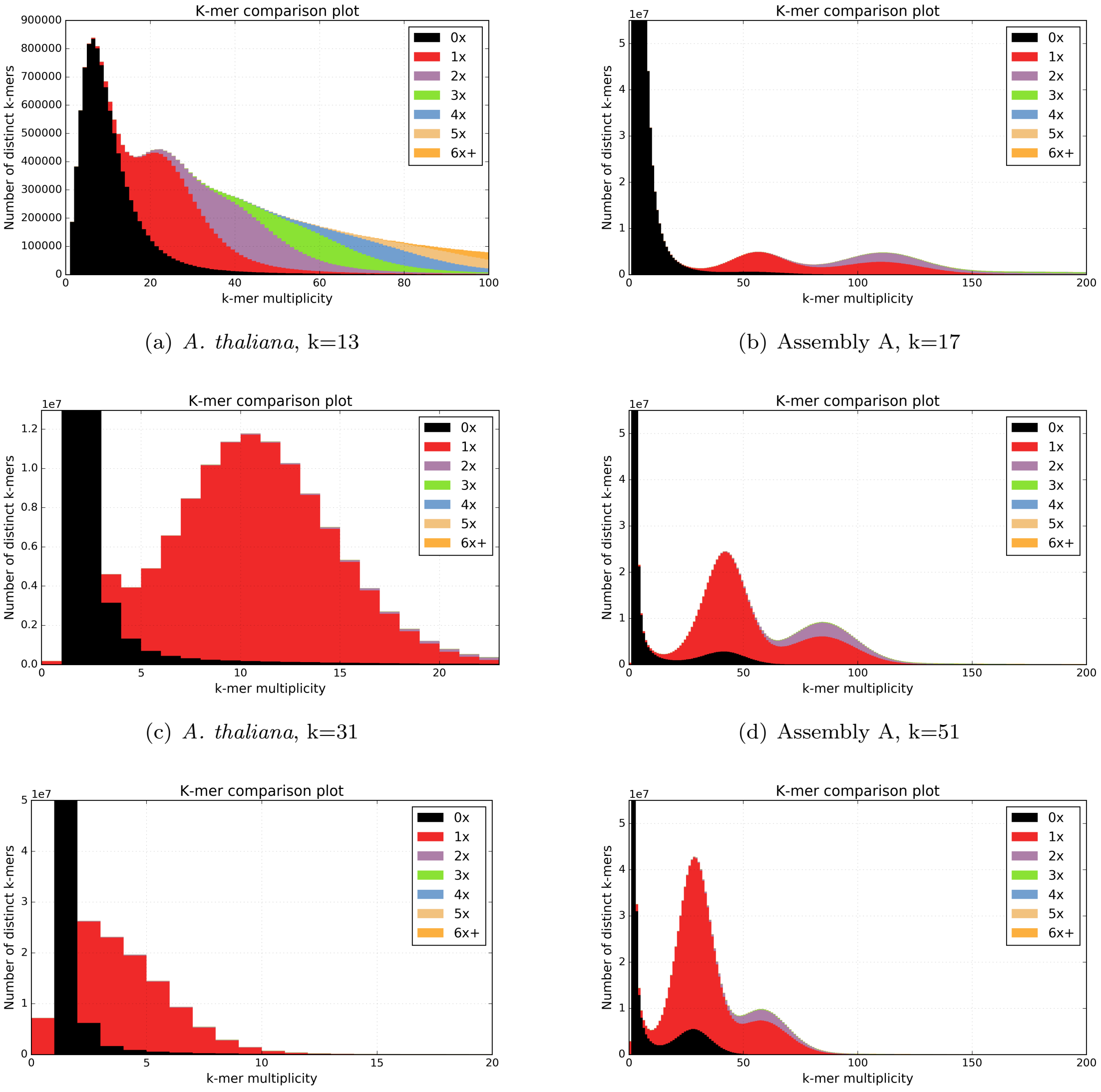

(a) *A. thaliana*, k=13

(b) Assembly A, k=17

(c) *A. thaliana*, k=31

(d) Assembly A, k=51

(e) *A. thaliana*, k=71

(f) Assembly A, k=101

Figure 1: Effect of k on a spectra-cn plot generated with KAT *comp*. The *A. thaliana* example shows too small (a), correct (c) and too large (e) k values for the SRR519624 dataset. The *F. excelsior* example shows three different k values in the correct range for this dataset (i.e. the range at which the distributions are easy to appreciate, with enough coverage and not collapsing into one another) and how they affect the relative abundance of elements in the distributions and their frequencies. Axis limits on this last example have been kept equal to showcase relative changes for different k.

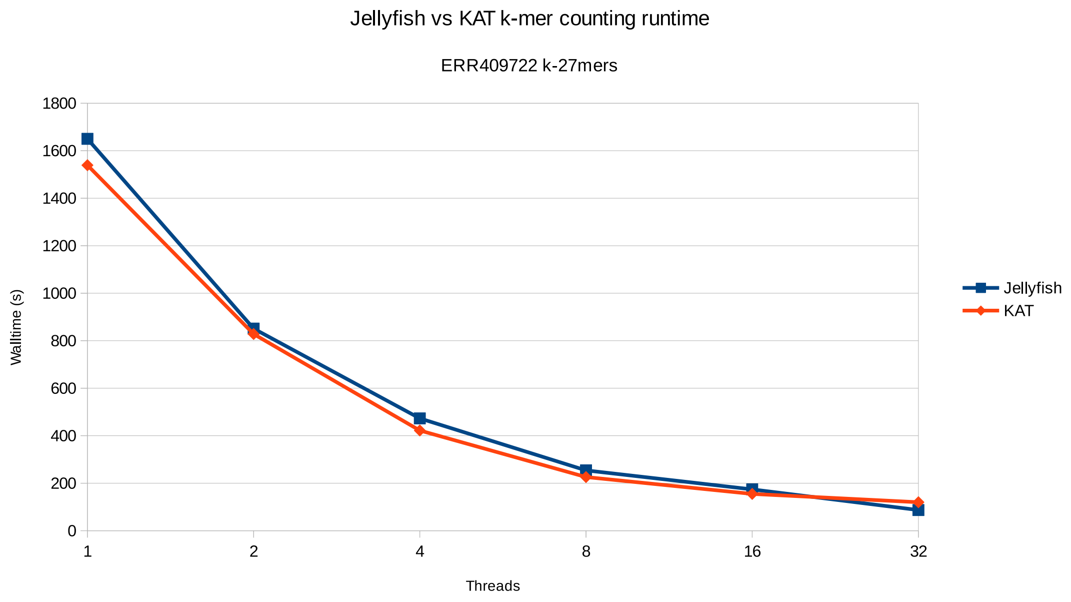

Figure 2: Comparison of k-mer counting runtimes from jellyfish V2.2.1 to KAT V2.1.0 at varying thread counts using Isilon scale out NAS. Command lines used: KAT - "kat hist -m 27 -H 500000000 -t <threads> -C -o kat-<threads>.hist ERR409722_?.fastq"; Jellyfish - "jellyfish count -m 27 -s 500000000 -t <threads> -C -o jellyfish-< threads>.jf27 ERR409722_?.fastq".

are identical between Jellyfish and KAT maximum memory usage in both cases is approximately 2.7GB. Also for both cases, the runtime speed up is almost linear when thread count is low. As thread count increases less performance per thread is achieved, due to IO bandwidth and other overheads within the code eventually become a limiting factor.

4.2 K-mer counting compared to kPAL

In Supplementary Figure 4, we compare k-mer counting runtimes from kPal V2.2.1 to KAT V2.0.6 varying k-values. Because kPAL does not support multi-threading we set KAT to use only a single thread and larger k values. Despite this KAT outperforms kPAL in the kmer counting runtime, especially when considering the speed-up gained from multi-threading. In addition, Supplementary Figure 5 shows that kPAL requires more memory than KAT for equivalent k values, indicating that Jellyfish can store k-mers more compactly than kPAL. In addition, the primary reason kPAL cannot support larger k values is due to rapidly escalating memory usage as k increases. However, kPAL can process k-mers with very low k (lower than 12), where KAT fails.

4.3 Tool runtimes

Table 2 highlights the proportion of time spent during k-mer counting relative to k-mer processing in each KAT tool. Here we have chosen k-27 and 8 threads, and for *sect* we used an SPADES V3.5.0 (Bankevich *et al.*, 2012) assembly of ERR409722 as the target reference, which contained 724 scaffolds. Supplementary Figure 6 shows how KAT tools scale with the number of threads. The

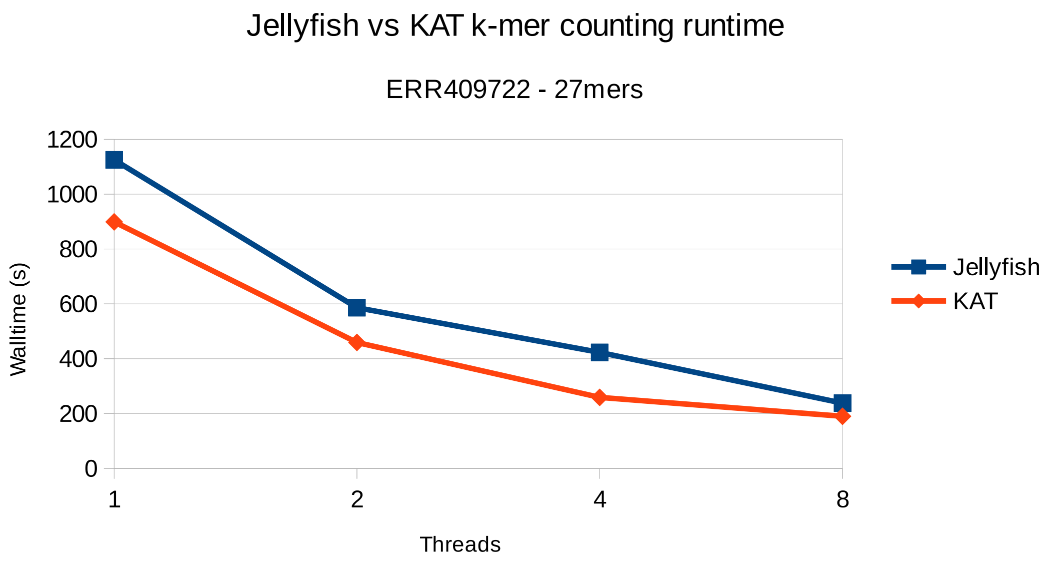

Figure 3: Comparison of k-mer counting runtimes from jellyfish V2.2.1 to KAT V2.0.6 at varying thread counts using SSD storage. For these tests a machine with an Intel i7 930 CPU with 12GB RAM was used, executing the same command lines as Supplementary Figure 2.

Method	Counting (s)	Processing (s)	% time processing
hist	226	6	2.6
gcp	259	19	6.8
comp	297	53	15.1
sect	205	57	21.8

Table 2: Comparison of KAT counting vs k-mer processing, using 8 threads.

actual runtimes are dependent on the actual settings, files and environment used for each tool so the figure is purely meant to demonstrate scalability and not that one tool is faster or slower than another. *hist* and *gcp* tools scale linearly at least up to 32 threads, and should scale well beyond this. *comp* scales linearly until around 8 threads after which overheads attributed to merging results from each thread come start to become a significant factor. For the *sect* tool we do not experience linear speedup proportionate to the number of threads, although gains are experienced until 16 threads, at which time the process becomes IO bound on our isilon storage system. While these results are derived from one dataset, one k-mer setting and are specific to our hardware, we expect the overall trends to hold across datasets and environments.

5 Assembly analysis and validation using k-mer spectra

The assembly spectra copy number plot, produced by the *comp* tool, provides information to analyse how much and what type of k-mer content from the reads makes it into the assembly. It decomposes

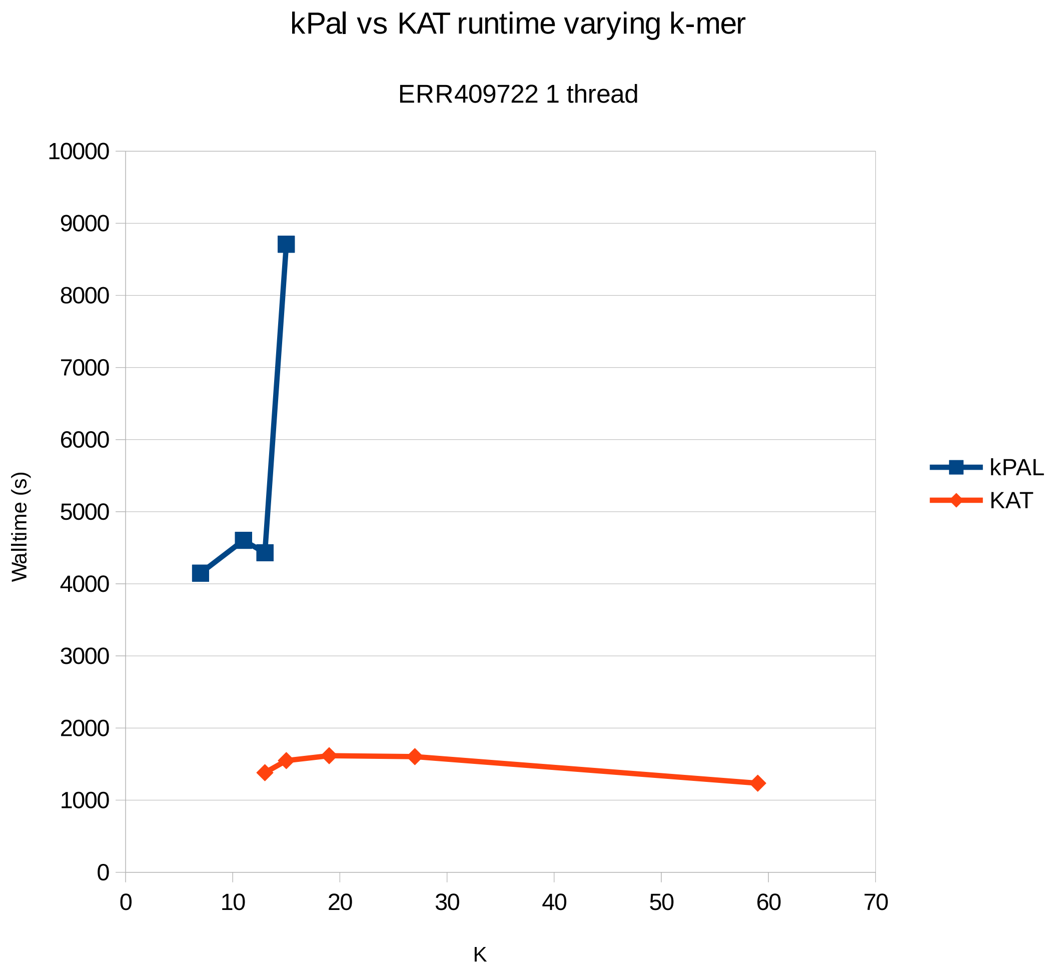

Figure 4: Walltime comparison of k-mer counting from KAT V2.1.0 to kPAL V2.2.1 at varying k values.

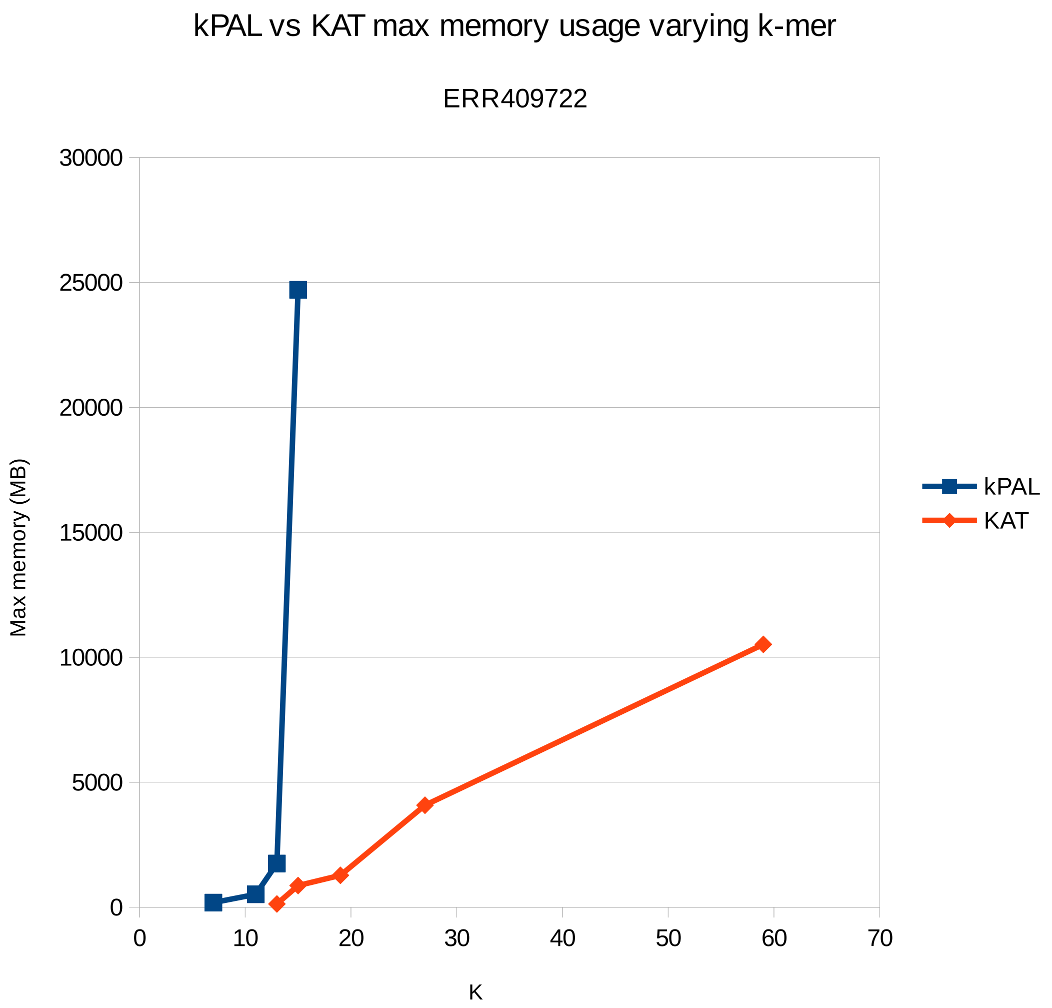

Figure 5: Maximum memory usage comparison of k-mer counting from KAT V2.1.0 to kPAL V2.2.1 at varying k values.

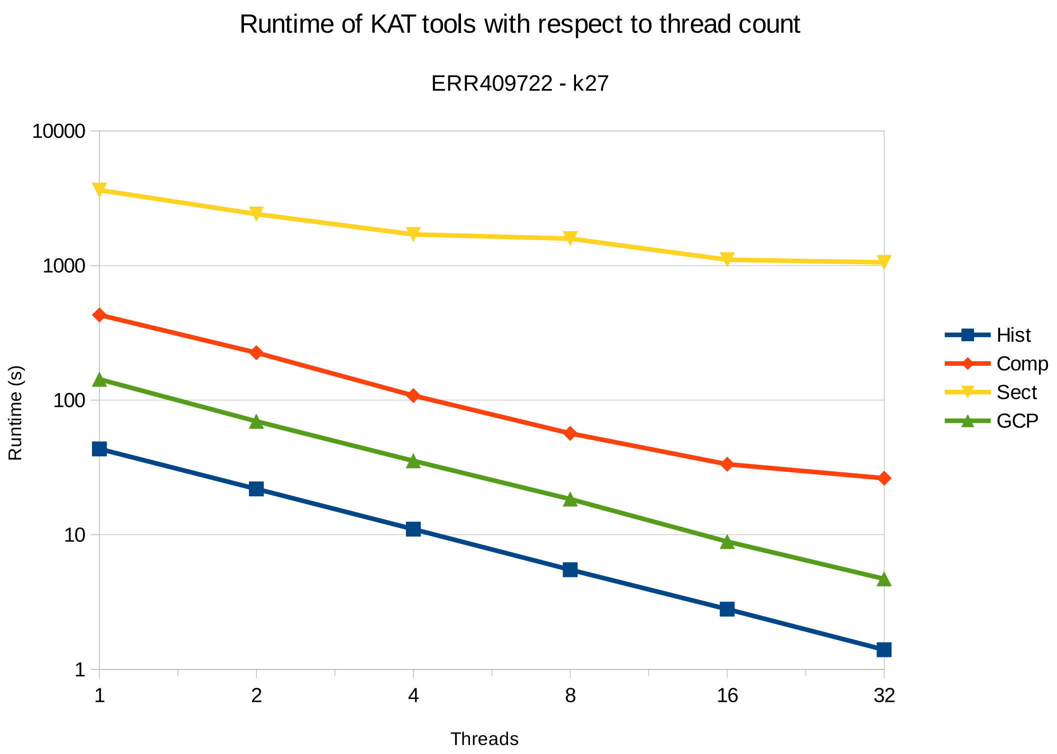

Figure 6: Scalability of KAT tools with number of threads (k-mer counting excluded from runtime).

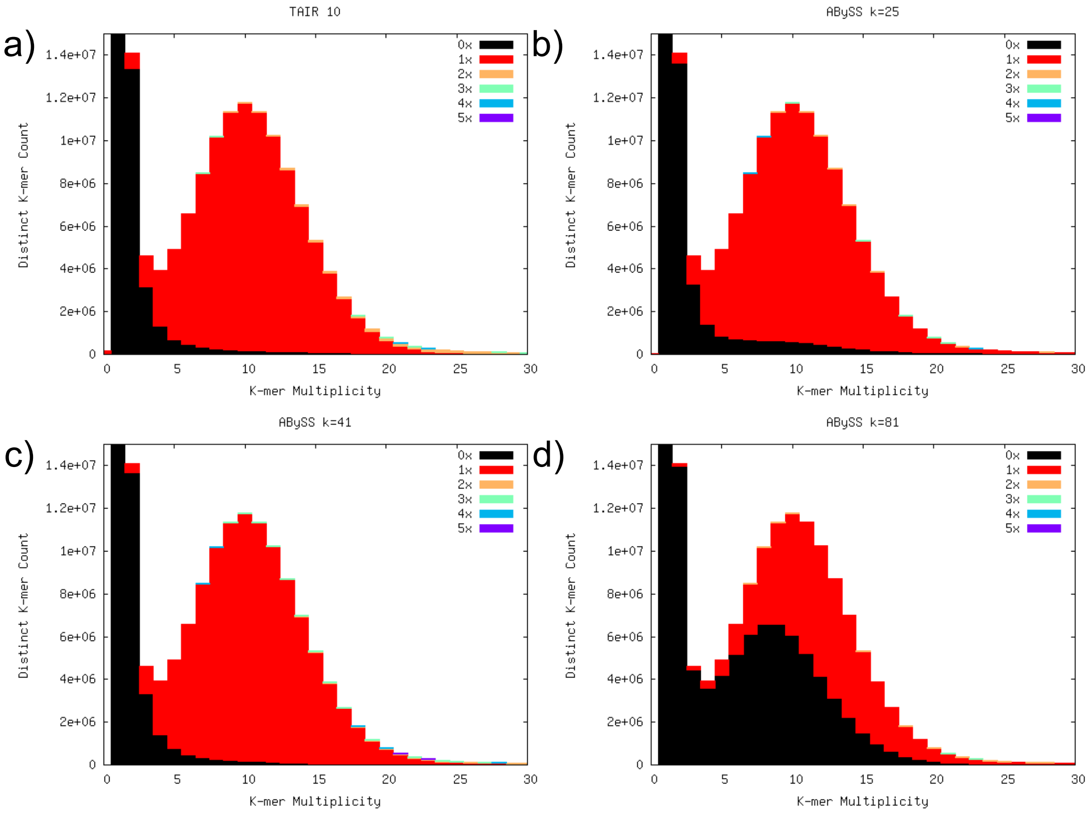

Figure 7: K-mer spectra copy number plots. Colour on the stacked bars represents copy number on the assembly, frequency counts (spectral distribution) are computed on the reads. a) Reference assembly for A.thaliana Columbia-0 versus a real sequencing run (SRR519624). b) ABySS(Simpson *et al.*, 2009) assembly at k=25 c) ABySS assembly at k=41 d) ABySS assembly at k=71

the k-mer spectrum of a read dataset by the frequency in which the k-mers are encountered in the assembly. Put another way, we represent how many elements of each frequency in the reads spectrum ended up included only once in the assembly, how many twice, etc. In addition, we record how many k-mers do not appear in the assembly. If an assembler is performing well, sequencing errors are generally absent from the assembly, the genuine unique content is there exactly once (for haploid genomes), and repeated content at higher levels of duplication with distributions centred around multiples of the sampling frequency for unique content.

Across different datasets and species, we generally observe different degrees of reconstruction for the different components of the spectrum. Supplementary Figure 7 shows the analysis for different assemblies over the same *A. thaliana* dataset (SRR519624). The histograms corresponding to absent k-mers (black) indicate the assembly performed by DeBruijn Graph at k = 41 (7c) includes more content from the dataset than those with k = 25 (7b) and k = 71 (7d), but still falls short of including all content present in the reference assembly (7a). This analysis highlights the level of assembly completeness: a lower number of distinct k-mers absent in the reference at the frequency of average sampling depth indicates a more complete assembly.

The spectra-cn plot also shows expected contractions of heterozygous content and the effects

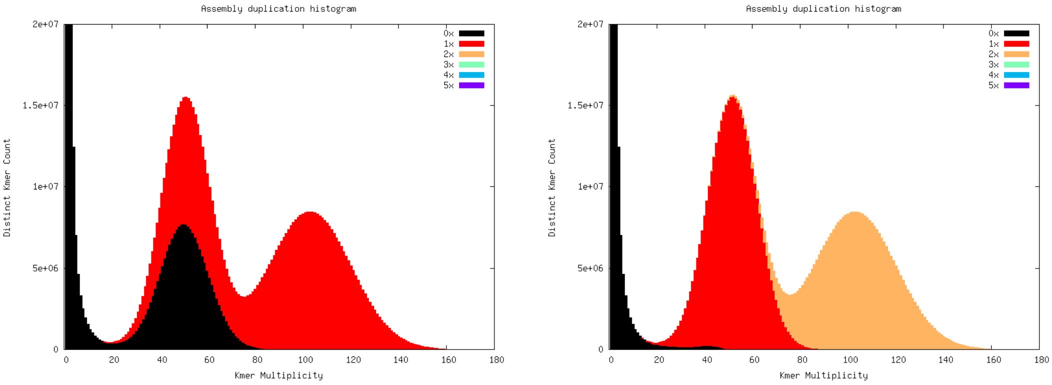

(a) Single haplotype

(b) Separated haplotypes

Figure 8: Perfect heterozygous assemblies

of polyploidy where the decomposed distributions generated using the assembly frequencies are inconsistent with the read spectrum. Heterozygous genomes produce more interesting and complex plots, since their k-mer spectra clearly shows different distributions for both the heterozygous and the homozygous content. Supplementary Figure 8 shows what a good diploid assembly looks like. In 8a we have a single haplotype mosaic, where the bubbles are collapsed, which is what we typically expect as correct from most illumina-based assemblers, although it is worth noting that alternate alleles are lost when we do this. In 8b haplotypes are separated producing the duplication of content in the homozygous regions, allowing the full representation of heterozygous content. Normally, we dont aim for the second scenario when assembling genomes. Interestingly though, in practice, assemblers of real heterozygous samples tend to retain duplications and include extra variation where it is not possible to assemble through heterozygous regions, as shown in Supplementary Figure 9.

A more detailed explanation about how the extra duplications appear on heterozygous assemblies such as the one on Figure 1 of the Main text, can be seen in the example loci shown for them. Supplementary Figure 10 show the full set of 3 copies for assembly A and 2 copies for assembly B.

Assembly B reconstructs 2 loci that include the heterozygous duplicated elements presenting the peaks. Each one of this two loci is reconstructed once, as shown in (b) and (d) by the Kmer coverage in the assembly being 1, except for the repeated-duplication peaks. Even locus 2, which contains heterozygous content (as shown by the read coverage halving after position 400), is assembled only once, with one of the 2 haplotypes assembled and the other being discarded.

Assembly A, on the other hand, creates a second copy of locus 2. This is a typical behaviour of assemblers when some heterozygous content create extra paths on the assembly graph to represent al the content. In this, however, not only a second copy of locus 2 is created, as shown in (c) and (e) by the main assembly coverage being 2, but also the haplotype information is being incorrectly represented, given that we end up with 2 copies of the same haplotype, which can be seen on the coverage for the assembly continuing to be 2 on the region corresponding to the halving of the read coverage after 400. The whole of locus2 will then contribute on the Main text's Figure 1 (a) plot to the duplications seen on the heterozygous and homozygous distributions, and to triplications of duplicated-frequency (i.e. 200) content.

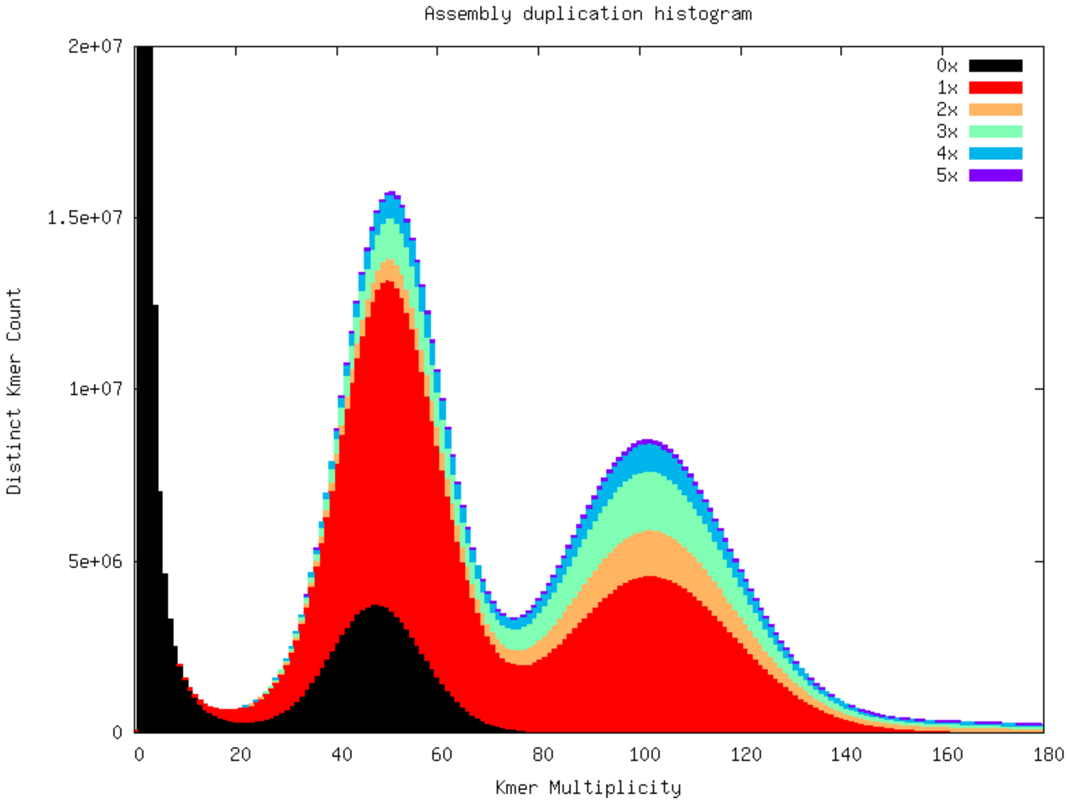

Figure 9: A real assembly of a heterozygous WGS dataset.

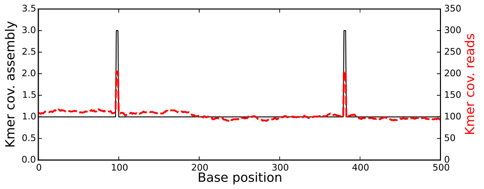

(a) Assembly A, locus 1

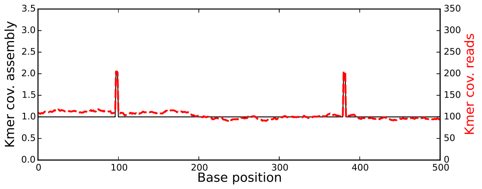

(b) Assembly B, locus 1

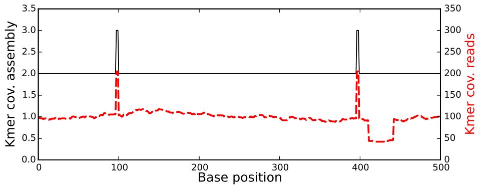

(c) Assembly A, locus 2 (expanded, copy #1)

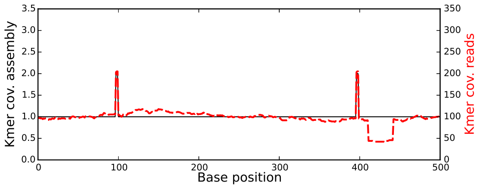

(d) Assembly B, locus 2 (collapsed)

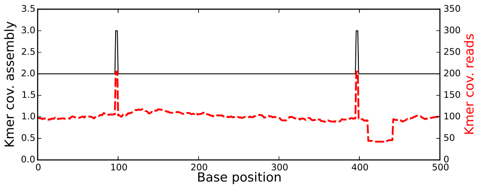

(e) Assembly A, locus 2 (expanded, copy #2)

Figure 10: K-mer coverage across all different assembled sequences for the loci mentioned on Figure 1 on the Main Text, showing an extra copy for the heterozygous locus on assembly A (copies on (d) and (e)) and correct haplotype collapsing on assembly B (c).

References

Anvar, S.Y. et al (2014). Determining the quality and complexity of next-generation sequencing data without a reference genome. *Genome Biol*, **15**(12), 555.

Bankevich, A. et al (2012). Spades: a new genome assembly algorithm and its applications to single-cell sequencing. *J Comput Biol*, **19**(5), 455–477.

Simpson, J.T. (2014). Exploring genome characteristics and sequence quality without a reference. *Bioinformatics*, **30**(9), 1228–1235.

Simpson, J.T. et al (2009). Abyss: a parallel assembler for short read sequence data. *Genome research*, **19**(6), 1117–1123.
